# Descriptive Analysis of Oxygen Dependency and Hemodynamic Changes Following Pulmonary Thromboendarterectomy in Chronic Thromboembolic Pulmonary Hypertension Patients

**DOI:** 10.7759/cureus.77663

**Published:** 2025-01-19

**Authors:** Jason N Patel, Andrew Talon, Chengcheng Hu, Mariel Ma, Sailendra Chundu, Taylor Burton, Orazio Amabile, Nafis Shamsid-Deen, Karim El-Kersh, Abdul Khan

**Affiliations:** 1 College of Medicine, University of Arizona College of Medicine, Phoenix, USA; 2 Pulmonary and Critical Care Medicine, University of Arizona College of Medicine, Phoenix, USA; 3 Biostatistics, University of Arizona College of Medicine, Phoenix, USA; 4 Internal Medicine, University of Arizona College of Medicine, Phoenix, USA; 5 Cardiothoracic Surgery, University of Arizona College of Medicine, Phoenix, USA

**Keywords:** cardiothoracic and vascular surgery, chronic thromboembolic pulmonary hypertension (cteph), hemodynamic measurements, pulmonary endarterectomy, surgical outcomes research, treatment of pulmonary hypertension

## Abstract

Background

Chronic thromboembolic pulmonary hypertension (CTEPH) is characterized by persistent clot burden and secondary vascular remodeling. Pulmonary thromboendarterectomy (PTE) remains the preferred curative treatment for CTEPH. This study presents a descriptive analysis of surgical outcomes in patients undergoing PTE for CTEPH at a tertiary referral hospital.

Methods

From 2015 to 2023, 40 CTEPH patients underwent PTE. Data on perioperative and postoperative outcomes were retrospectively collected. Continuous variables were compared between two independent groups by the Wilcoxon rank-sum test, and the Wilcoxon signed-rank test was used for comparison of pre-op and post-op variables of the same patient.

Results

There was an overall improvement in postoperative hemodynamics. Postoperative hemodynamics were not affected by surgical classification of disease level (59.5% of cases were Level 2 lobar lesions). After a median follow-up of three years, the overall survival rates were 0%, 10%, and 7.5%, respectively. Patients unable to discontinue oxygen at discharge had longer median circulatory arrest times (44.5 versus 23 minutes; p=0.019). Patients requiring at least 1 L/min of oxygen preoperatively had a longer hospital length of stay (LOS) than patients without oxygen requirements (25 versus 10 days; p=0.019). Lower tricuspid annular plane systolic excursion (TAPSE) (p=0.0013), higher right atrial pressure (p=0.0013), and higher mean pulmonary artery pressure (mPAP) (p=0.048) preoperative values were also associated with increased LOS.

Conclusions

PTE improves hemodynamic parameters in patients with CTEPH, with an acceptable safety profile. Clinicians must identify risk factors accurately in order to weigh the benefits and risks of surgery and help intensivists identify high-risk patients. There are certain risk factors that may require closer hemodynamic monitoring, which could improve prognosis by personalizing management strategies. Future research should focus on optimizing patient selection criteria through predictive modeling, refining surgical techniques to reduce circulatory arrest times, and exploring the long-term benefits of adjunctive therapies. The hypothesis that patients without the need for supplemental oxygen have shorter hospital stays after PTE is supported by the physiological basis that these patients are less likely to experience severe reperfusion injury, have less significant microvasculopathy, and exhibit better hemodynamic stability. In contrast, patients requiring oxygen may face more complex postoperative challenges, necessitating longer hospital stays for adequate management and recovery.

## Introduction

Chronic thromboembolic pulmonary hypertension (CTEPH)

CTEPH is a subgroup of pulmonary hypertension (PH) (World Health Organization (WHO) group 4) caused by endothelialization and obstruction of the pulmonary arteries, resulting in increased pulmonary vascular resistance (PVR) and right ventricular (RV) failure [1]. Small vessel vasculopathy and plexiform lesions develop over time, which is the same pathology seen in WHO group I pulmonary arterial hypertension (PAH) [1].

A meta-analysis of 16 studies involving 4,047 patients found that 0.56%-6.3% of acute PE cases result in CTEPH [2]. CTEPH only occurs in a subset of patients for a variety of reasons, including recurrent injury or underlying hypercoagulable states [3-5]. Antiphospholipid syndrome, splenectomy, and female sex have also been identified as additional risk factors [3-5].

PAH is characterized by exercise intolerance, dyspnea, fatigue, volume overload, and hypoxia, which are nonspecific symptoms that contribute to its under recognition and negative impact on prognosis, with an average delay of 14 months between diagnosis and symptom onset [4].

Chronic obstruction of pulmonary arteries by organized thrombi can occur with or without evidence of clinical PH. Isolated chronic obstruction on imaging without evidence of a clinical syndrome of PH is referred to as chronic thromboembolic disease (CTED). A ventilation-perfusion (V/Q) scan is the screening method of choice for CTEPH/CTED with a sensitivity of 90-100% and specificity of 94-100% compared to computed tomographic pulmonary angiography (CTPA) [6]. Multiple segmental or subsegmental mismatches on V/Q scan are indicative of CTEPH/CTED. CTPA findings include RV hypertrophy, bronchial artery collaterals, mosaic attenuation, and pulmonary artery abnormalities (webs, pouch defects, recanalization, narrowing of distal vessels/stenosis) [6]. The definitive diagnosis of CTEPH/CTED requires pulmonary artery angiography with right heart catheterization (RHC). The hemodynamic definition of CTEPH is identical to that of precapillary PH: mean pulmonary arterial pressure (mPAP) greater than 20 mmHg, PVR greater than 2.0 Wood units (WU), and pulmonary capillary wedge pressure (PCWP) less than 15 mmHg [7]. Concurrent pulmonary artery angiography allows for detailed visualization of pulmonary arteries, identifying if obstructions are in proximal locations that are reachable by surgery. 

Pulmonary thromboendarterectomy (PTE) remains the preferred and potentially curative option for CTEPH [1]. Earlier referral to a CTEPH center is also associated with decreased mortality as specialists can assess surgical candidacy through further imaging studies such as cardiac magnetic resonance [1].

Eligibility for PTE

Comorbidities, hemodynamics, and clot accessibility all play a role in surgical selection. PTE is generally effective for main, lobar, or proximal segmental pulmonary artery branches. The results of a recent international prospective registry of 679 patients with CTEPH concluded that 36% were inoperable [4]. Among these patients, 48% were inoperable due to distal clot burden, 10% due to discordance between accessible clot burden and preoperative PVR, and 4% due to severely elevated PVR (>18.75 WU) [4]. In the registry, surgical candidates were not different from poor candidates in terms of symptom severity or NYHA functional classification [4].

In patients who are ineligible for PTE or have residual PH after PTE, balloon pulmonary angioplasty (BPA) is an alternative in expert centers [8]. BPA is a minimally invasive procedure that involves the use of a balloon catheter to dilate narrowed or blocked pulmonary arteries. Studies have shown that BPA can be safely performed after PTE and can further reduce PVR and improve cardiac output [8]. For others, pulmonary vasodilators are typically used as salvage therapy. Riociguat is the most reliable pulmonary vasodilator in this cohort. Riociguat stimulates soluble guanylate cyclase independent of nitric oxide, resulting in vasorelaxation [8]. Other less robust data include Macitentan, an endothelin receptor antagonist, and Treprostinil, a prostacyclin analogue [9-10].

Safety of PTE

PTE is considered a safe surgery and is highly correlated with surgical center volume. A systematic review totaling 9,763 patients across 55 single centers between 1970-2021 reported inpatient mortality rates of <3.5% in a center performing >50 PTEs/year, 4.5% with 11-50 PTEs/year, and 8.8% with <10 PTEs/year [11-12].

PTE involves administering cardioplegia, cardiopulmonary bypass (CPB), and circulatory arrest with deep hypothermia [11-12]. In a study of 338 PTE surgeries, the average CPB time was 215.8±47.0 minutes, and the average circulatory arrest time was 42.8±14.5 minutes [11-12]. Neither were predictors for in-hospital or follow-up mortality. This is also in agreement with another study of 122 patients that showed neither CPB time nor circulatory arrest time could predict in-hospital mortality, mortality within one month after PTE, or major adverse cardiac events long term [13-14]. However, in other studies looking at outcomes of thoracic surgeries requiring CPB and circulatory arrest, such as aortic dissections or aneurysms, authors have found that prolonged circulatory arrest time was associated with increased mortality [15-16]. CPB time greater than 200 minutes was also found to be a risk factor for postoperative severe systemic inflammatory response syndrome in those undergoing total aortic arch replacement [15-16].

Efficacy of PTE

Studies have shown that PTE improves both patient- and disease-oriented outcomes. A study of 40 patients with CTEPH found a nearly two-fold increase in 6 Minute Walk Test (6MWT) distance from 216.6 meters to 410.5 meters after PTE [17], while another study of 110 patients (51 with pre- and postoperative 6MWT) demonstrated an increase in 6MWT distance from 378 meters to 410 meters [13, 17]. Based on a 36-item medical outcomes study/rand survey, researchers found that quality of life scores after PTE were comparable to those from age-matched, healthy controls without CTEPH [17]. From a hemodynamic standpoint, several studies report improvements in mPAP and PVR [18]. Moreover, PTE is associated with statistically significant three-year mortality benefit, with one study citing 89% survival in the PTE group versus 71% survival in the medical therapy group [18-19].

## Materials and methods

This descriptive analysis examines the surgical outcomes of patients who underwent PTE for CTEPH between January 1, 2015, and December 31, 2023. This manuscript was part of an IRB-approved study by the University of Arizona College of Medicine Phoenix, with ethics approval indicating that, as a retrospective study without patient identifiers, no consent was required. Patients were identified using the International Classification of Diseases (ICD-10) code data. To ensure the completeness and accuracy of the pooled data, several validation steps were undertaken. Extracted ICD-10 codes were cross-verified with patient records by our biostatistician to confirm all relevant cases were included. Diagnosis of CTEPH was confirmed based on imaging and invasive hemodynamic criteria when available. The dataset underwent a rigorous cleaning process where duplicated entries were removed, and any discrepancies were addressed. Dataset was routinely manually reviewed to ensure that the ICD-10 codes accurately reflected the patients' diagnoses and that no relevant data was omitted. Data collected from medical charts included demographics (age, sex, BMI, WHO functional class for PH), comorbidities, oxygen requirements, N-terminal prohormone brain-type natriuretic peptide (NT-proBNP), 6MWT, hemodynamic parameters (RHC, echocardiogram), and imaging results (V/Q scans, CTPA). Data was also collected on PTE procedural parameters such as circulatory arrest and CPB times, postoperative medical management strategies, and LOS. Patient selection criteria, including specific characteristics for PTE inclusion, were detailed to clarify the study's scope.

The patient’s subjective functional assessment was performed in accordance with the standard WHO definitions for functional classes I-IV. The University of California, San Diego (UCSD) surgical classification of pulmonary occlusive disease was utilized. In this system, Level 0 indicates no surgical evidence of chronic thromboembolic disease, while Level 1 refers to obstruction in a main pulmonary artery. Level 2 involves the main descending pulmonary arteries or lobar arteries, Level 3 is characterized by the involvement of segmental arteries, and Level 4 refers to distal (subsegmental) lesions [20].

Surgery was performed using the method established by the UCSD group [20]. A median sternotomy was performed, extracorporeal circulation was established through the ascending aorta and the superior vena cava, and blood was cooled until the core body temperature reached 18°C. After clamping of the ascending aorta and cardioplegic solutions were infused, an endarterectomy was performed. Systemic circulation was restored after the endarterectomy, and the incision was closed while normalizing body temperature. 

Study data was collected and managed using REDCap electronic data capture. Count and percentage was used for categorical variables. Continuous variables were compared between two independent groups by the Wilcoxon rank-sum test, and the Wilcoxon signed-rank test was used to compare the pre-op and post-op variables of the same patient. A p-value of <0.05 was considered statistically significant. The choice of statistical tests was based on data distribution assessments, with non-parametric tests selected due to non-normal distributions. Data management protocols, such as validation and handling of missing data, were implemented to enhance reliability. Small sample sizes for certain comparisons were acknowledged. The statistical analysis was supported by the University of Arizona College of Medicine Phoenix Biostatistics and Study Design Services.

## Results

Demographics and comorbidities

A total of 40 patients met the inclusion criteria. All demographics and preoperative characteristics are shown in Table 1. The average age at diagnosis was 50.9 years. The average time between diagnosis and surgery was 36 days. Before PTE, 42.5% of patients were WHO Functional Class II. Of patients who underwent a preoperative V/Q scan, 93.3% had a perfusion defect. In 59.5% of cases, the disease was classified as Level 2 lobar lesions. All comorbidities are shown in Table 2 and Table 3. Pertinent comorbidities were anticoagulation use (97.5%), history of PE (90%), deep vein thrombosis (57.5%), presence of inferior vena cava filter (30%), and diabetes mellitus (25%). Six patients (15%) had thrombophilia diagnosis: two with Factor V Leiden mutations, one with antiphospholipid syndrome, one with antithrombin III deficiency, one with Protein S deficiency, and one with sickle cell disease.

**Table 1:** Demographics and preoperative characteristics. All quantitative variables are reported as median values. IQR: interquartile range; V/Q: ventilation/perfusion; WHO: World Health Organization.

Gender	Count (%)
Female	21 (52.5)
Male	19 (47.5)
Race	Count (%)
American Indian or Alaska native	1 (2.5)
Black or African American	8 (20)
White	30 (75)
Not Reported	1 (2.5)
Ethnicity	Count (%)
Hispanic or Latino	4 (10)
Not Hispanic or Latino	34 (85)
Unknown	2 (5)
Age	Years (IQR)
At diagnosis	50.9 (41.3, 61.7)
At surgery	51.1 (41.6, 62)
Time from diagnosis to surgery, days (IQR)	36 (12.8, 84.8)
Preoperative vitals	Value (IQR)
BMI	31.9 (25.8, 35)
Preoperative WHO functional class	Count (%)
Class I	7 (17.5)
Class II	17 (42.5)
Class III	14 (35)
Class IV	2 (5)
Preoperative V/Q scan perfusion defect	Count (%), n=30
No	2 (6.7)
Yes	28 (93.3)
Preoperative V/Q scan ventilation defect	Count (%), n=23
No	21 (91.3)
Yes	2 (8.7)
Preoperative CT angiogram level of disease	Count (%), n=37
Level 0	1 (2.7)
Level 1	22 (59.5)
Level 2	11 (29.7)
Level 3	3 (8.1)

**Table 2:** Comorbidities. All quantitative variables are reported as N and %.

Comorbidities	Count (%)
Anticoagulation use	39 (97.5)
Aspirin/plavix use	8 (20)
Asthma	7 (17.5)
Atrial fibrillation	6 (15)
Autoimmune disease	5 (12.5)
Cancer/Malignancy	7 (17.5)
Cerebral vascular accident/stroke	7 (17.5)
Chronic kidney disease	3 (7.5)
Chronic venous insufficiency	1 (2.5)
Congestive heart failure	7 (17.5)
Chronic obstructive lung disease	11 (27.5)
Coronary artery disease	4 (10)
Diabetes mellitus	10 (25)
Deep vein thrombosis	23 (57.5)
Family history of deep vein thrombosis or pulmonary embolism	6 (15)
Fracture	2 (5)
Human immunodeficiency virus	2 (5)
Hypertension	17 (42.5)
Interstitial lung disease	0 (0)
Inflammatory bowel disease	0 (0)
Cirrhosis	1 (2.5)
Major surgery	14 (35)
Nephrotic syndrome	0 (0)
Obstructive sleep apnea	4 (10)
Oral contraceptive use	0 (0)
Peripheral artery disease	0 (0)
Pregnancy	0 (0)
Presence of inferior vena cava filter	12 (30)
Pulmonary emboli	36 (90)
Splenectomy	0 (0)
Stimulant or anorexigen use	1 (2.5)
Thrombophilia diagnosis	6 (15)
Thyroid disorder	2 (5)
Varicose veins	2 (5)

**Table 3:** Frequency of PE and DVT Incidences. DVT: deep vein thrombosis; PE: pulmonary embolism.

Number of PE or DVT events	Number of patients with history of PE (%), n=36	Number of patients with history of DVT (%), n=23
1	19 (52.8)	11 (47.8)
2	11 (30.6)	9 (39.1)
3	4 (11.1)	1 (4.3)
4	2 (5.6)	2 (8.6)

Cardiac and hemodynamic markers

**Figure 1:**
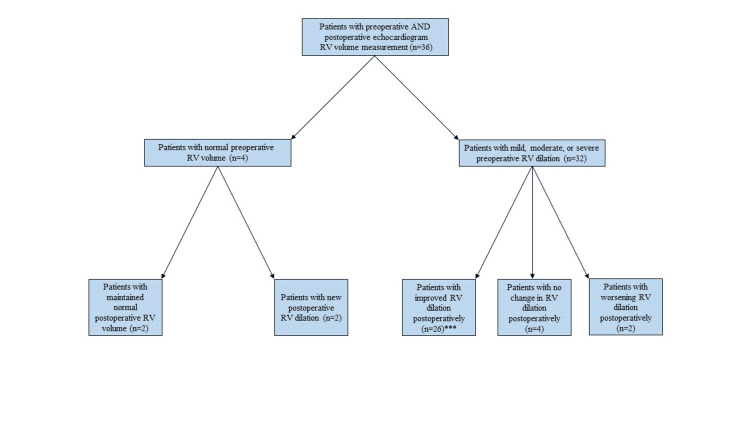
Flow diagram of change in right ventricle (RV) volume after PTE. ***p=0.003. Continuous variables were compared between two independent groups by the Wilcoxon rank-sum test, and the Wilcoxon signed-rank test was used for comparison of pre-op and post-op variables of the same patient. A p-value of <0.05 is considered statistically significant.

**Figure 2:**
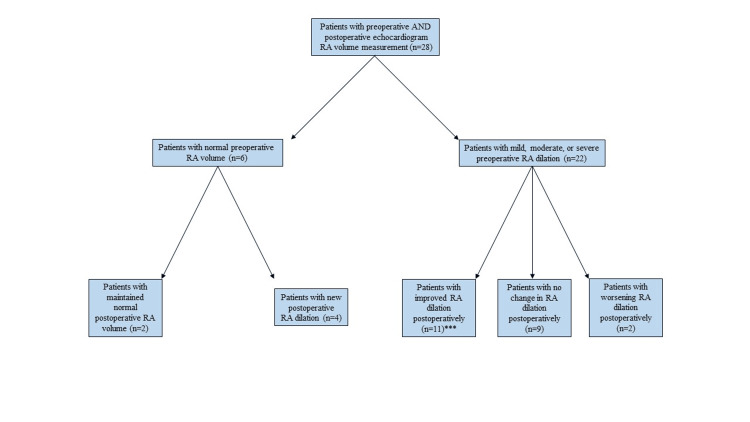
Flow diagram of change in right atrium (RA) volume after PTE. ***p=0.035. Continuous variables were compared between two independent groups by the Wilcoxon rank-sum test, and the Wilcoxon signed-rank test was used for comparison of pre-op and post-op variables of the same patient. A p-value of <0.05 is considered statistically significant.

After PTE, median right ventricular systolic pressure (RVSP) decreased from 74 mmHg (59.5, 85) to 45.5 mmHg (27, 55) (p=0.0002), tricuspid annular plane systolic excursion (TAPSE) increased from 13.5 mm (10, 17.4) to 15.1 mm (11.9, 20.2) (p=0.11), tricuspid peak velocity decreased from 3.9 m/s (3.5, 4.3) to 2.5 m/s (2.3, 3.2) (p<0.0001). PTE also resulted in a decrease in RV volume (p=0.0003) and RA volume (p=0.035) of at least one categorical value (Figure 1, Figure 2).

The majority of our patients were able to undergo a post-PTE echocardiogram. Right atrial pressure (RAP) decreased from 10 mmHg (4.5, 15) to 4 mmHg (3, 8), mPAP decreased from 46 mmHg (38.5, 50) to 26.5 mmHg (22.5, 46.8), and RHC PVR decreased from 7.2 WU (4.5, 9.1) to 3.6 WU (3.3, 4.2). Median preoperative NT-proBNP decreased from 2067.5 pg/mL (842, 4195.5) to 342.5 pg/mL (111.8, 1450.2) (p=0.0004). Median preoperative 6MWT distance increased from 288.32 meters (75, 332.84) to 358.4 meters (254.7, 390.1) (Table 4). 

**Table 4:** Preoperative and postoperative hemodynamic variables and cardiac markers. Continuous variables are presented as median (interquartile range) and categorical values as percentage. 6MWT: 6 minute walk test; mPAP: mean pulmonary artery pressure; NT-proBNP: N-terminal prohormone of brain natriuretic peptide; RAP: right atrial pressure; RHC: right heart catheterization; RVSP: right ventricular systolic pressure; TAPSE: tricuspid annular plane systolic excursion.

Variable	Preoperative value (IQR) (sample size)	Postoperative value (IQR) (sample size)	p-value	Number of patients with both pre-and postoperative data	Number of patients with improvement, (%)
Echocardiogram RVSP (mmHg)	74 (59.5, 85), (n=35)	45.5 (27, 55), (n=26)	0.0002	23	19 (82.6)
Echocardiogram TAPSE (mm)	13.5 (10, 17.4), (n=34)	15.1 (11.9, 20.2), (n=36)	0.11	30	27 (90)
Echocardiogram tricuspid peak velocity (m/s)	3.9 (3.5, 4.3), (n=26)	2.6 (2.3, 3.2), (n=28)	p<0.0001	23	21 (91.3)
RHC RAP (mmHg)	10 (4.5, 15), (n=24)	4 (3, 8), (n=14)	N/A	8	6 (75)
RHC mPAP (mmHg)	46 (38.5, 50), (n=31)	26.5 (22.5, 46.8), (n=10)	N/A	8	8 (100)
RHC PVR (WU)	7.2 (4.5, 9.1), (n=26)	3.6 (3.3, 4.2), (n=10)	N/A	6	4 (66.6)
NT-ProBNP (pg/mL)	2067.5 (842, 4195.5), (n=22)	342.5 (111.8, 1450.2), (n=20)	0.0004	14	13 (92.9)
6MWT (m)	288.32 (75, 332.84), (n=6)	358.4 (254.7, 390.1), (n=13)	N/A	3	2 (66.7)

**Figure 3:**
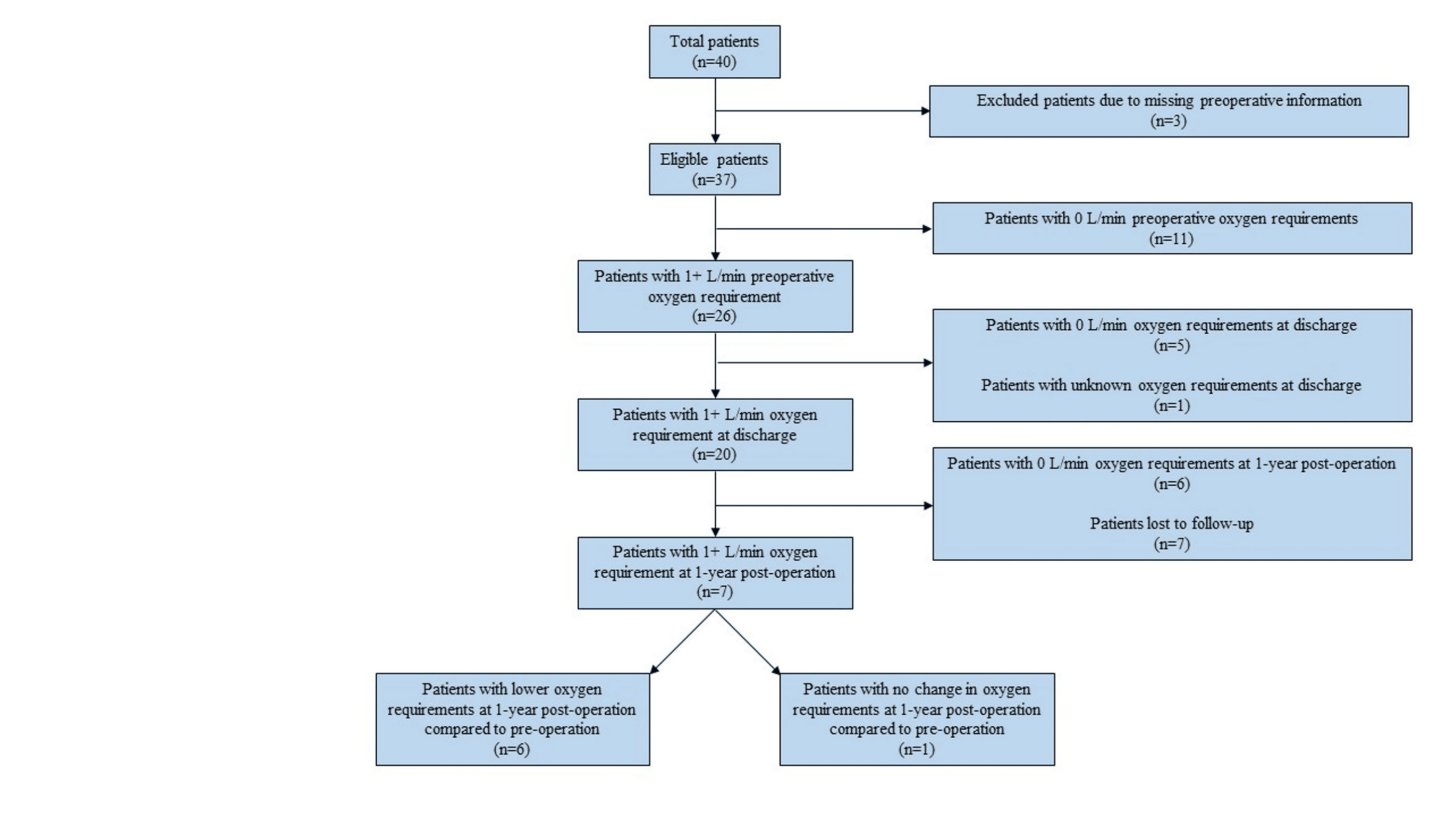
Flow diagram of study participants and their oxygen requirements at the preoperative, discharge, and 1-year postoperative time frames. Continuous variables were compared between two independent groups by the Wilcoxon rank-sum test, and the Wilcoxon signed-rank test was used for comparison of pre-op and post-op variables of the same patient.

Median preoperative oxygen requirements were 3.5 L/min (0, 3.5; n=37), discharge oxygen requirements were 2 L/min (1, 3; n=38) (p=0.0411 versus preoperative), and 1-year postoperative requirements were 0 L/min (0, 1; n=30) (p=0.000025). Figure 3 shows a schematic of the number of patients with oxygen requirements over the preoperative and postoperative course.

Surgical variables

Median PTE procedure, circulatory arrest, and CPB time were 248, 43, and 175 minutes, respectively (Table 5). The rate of patients on intraoperative vasodilatory therapy (inhaled epoprostenol, inhaled nitric oxide) was 22/40 (55%), and postoperatively was 28/40 (70%). In 87.5% of cases, diuretics were administered postoperatively. A median circulatory arrest time of 44.5 minutes (34.2, 47.2) was reported in those unable to discontinue oxygen at discharge versus 23 minutes (21, 34) in those who could discontinue oxygen at discharge (p=0.019).

**Table 5:** Surgical variables. All quantitative variables are reported as median values. CPB: cardiopulmonary bypass; ECMO: extracorporeal membrane oxygenation; IVC: inferior vena cava.

Intraoperative variables	Minutes (IQR)
Total procedure time	248 (213, 265), n=39
Total circulatory arrest time	43 (31.5, 46.2), n=36
Total CPB time	175 (159.5, 199), n=40
Intraoperative management	Count (%)
Intraoperative inhaled vasodilators	
No	18 (45%)
Yes	22 (55%)
Postoperative management	Count (%)
Steroids	
No	22 (55%)
Yes	18 (45%)
Diuretics	
No	5 (12.5%)
Yes	35 (87.5%)
Inhaled vasodilators	
No	12 (30%)
Yes	28 (70%)
IVC filter	
No	39 (97.5%)
Yes	1 (2.5%)
ECMO	
No	38 (95%)
Yes	2 (5%)

Factors affecting hospital length of stay

The median LOS was 21.5 days (9, 39). In patients with no preoperative oxygen requirements, the median LOS was 10 days (7, 16.5), and in patients with at least 1 L/min preoperative oxygen requirement, LOS was 25 days (p=0.019). Lower TAPSE (p=0.0013), higher RAP (p=0.0013), and higher mPAP (p=0.048) preoperative values were also associated with increased LOS.

Factors affecting mortality

Survivors were followed for a median of 723 days. 30-day, 1-year, and 3-year mortality rates were 0%, 10%, and 7.5%, respectively. The overall mortality rate was 17.5%, with a median time from surgery to death of 212 days. There was no significant association between preoperative oxygen requirements, WHO functional class, level of disease, echocardiogram or RHC hemodynamic variables, NT-proBNP, procedure time, circulatory arrest time, or CPB time on mortality (Table 2).

## Discussion

Cardiac and hemodynamic markers

We observed hemodynamic improvements in RVSP, tricuspid peak velocity, RV and RA volume, TAPSE, RAP, mPAP, and PVR after PTE. Our findings are overall consistent with previous studies and indicate restoration in right ventricular-pulmonary artery coupling (RV-PA) [11]. Successful PTE can reduce pulmonary vascular resistance, leading to a decrease in RV afterload. As a result, a reduction in RV volume post-surgery is often associated with improved RV function and better clinical outcomes [18-19]. An enlarged RA is typically a response to chronic RV pressure overload and can indicate the severity of right heart strain. It is often associated with atrial arrhythmias and can contribute to further hemodynamic instability. Reduced RV and RA volumes post-surgery are generally associated with improved symptoms, enhanced exercise capacity, and better overall survival rates. Studies show that these improvements are sustained over the long term, with most patients transitioning from WHO functional class III or IV preoperatively to class I or II postoperatively [17]. Post-PTE, our study also demonstrated improved 6MWT distances.

Our study did not find any association between the preoperative level of disease (59.5% had Level 2 lobar lesions) and postoperative hemodynamics. However, the complexity of thromboembolic disease does have a significant impact on outcome, highlighting the importance of patient selection once again. As compared to patients with more proximal thromboembolic disease (Levels 1 or 2), patients with distal pulmonary vasculopathy (Levels 3 or 4) had a higher postoperative tricuspid regurgitation, a higher PASP and a higher PVR [18-19].

Oxygen requirements and surgical technique

The oxygen requirements of patients decreased at discharge, and many discontinued oxygen therapy within one year. According to Tanabe et al., improvements in gas exchange capacity take 6-24 months after PTE, whereas hemodynamic improvements appear shortly after PTE [21]. There are several reasons for the delay, including impaired diffusion due to V/Q mismatch from pulmonary microvasculopathy, reperfusion pulmonary edema, postoperative atelectasis, and an increase in intrapulmonary shunts [21]. Prior studies found no correlation between CBP times or circulatory arrest times with postoperative outcomes. However, our findings suggest that patients may benefit from increased surgical efficiency by reducing intraoperative circulatory arrest times, which may reduce their need for oxygen postoperatively. In a prior study of patients undergoing resection of aortic arch aneurysms, cardiac dysfunction and respiratory complications were less common in those patients whose circulatory arrest times were shorter [15-16]. Of note, our average procedure time was 248 minutes, markedly lower than the 420 minutes reported in a recent study of 122 surgeries [22]. Median circulatory arrest time was 43 minutes (31.5, 47.2), and median CPB time was 175 minutes (159.5, 199), which is much shorter than the reported times of Sakurai et al., who reported total circulatory arrest time of 138 minutes (87, 207) and CPB time of 248 minutes (185, 456) [14]. It is well documented in post-cardiac arrest syndrome literature that hypoxia resulting from prolonged circulatory arrest may be multifactorial: ischemia-reperfusion injury, systemic inflammation, reperfusion pulmonary edema, and endothelial activation and coagulopathy [23]. The injury activates an inflammatory response by releasing reactive oxygen species and pro-inflammatory cytokines (TNF-alpha, IL-6, IL-8, IL-1), which leads to increased vascular permeability, leukocyte demargination, and extravasation [23]. Strategies to mitigate the risk associated with intraoperative circulatory arrest times could include optimizing anesthesia management, ensuring efficient surgical techniques, and using adjunctive therapies like ECMO support when necessary.

Complications

Our study reported no cases of postoperative bleeding or infections, reflecting effective surgical and perioperative management, consistent with findings from larger datasets. Extracorporeal membrane oxygenation (ECMO) was required in two patients (5.5%): one received veno-arterial ECMO for five days, and the other received veno-venous ECMO for 11 days. The incidence of postoperative respiratory failure requiring ECMO in our study aligns with a retrospective study of 127 patients, where 5.5% required ECMO [24], and is notably lower than a retrospective study of 42 patients, which reported an ECMO requirement in 26.6% of cases [24]. ECMO rates, compared to other studies, may vary due to differences in patient selection and institutional resources. 

Hospital stay

The median LOS in our study was 21.5 days, which is higher than the results of a prior study with 1398 patients where the median LOS was 15 days [11]. A lower TAPSE, higher RAP, and higher mPAP postoperatively were associated with longer hospital stays. A higher mPAP indicates more severe pulmonary hypertension, which is a direct measure of the pressure overload on the RV. Patients with higher mPAP preoperatively may have more extensive pulmonary vascular disease, potentially leading to longer recovery times and hospital stays. TAPSE is a marker of RV function. Lower TAPSE values typically indicate poorer RV function, while higher values suggest better RV function. However, in the context of CTEPH, even if TAPSE value is higher, the RV may still be compromised.. Patients with higher TAPSE but still within a pathological range might experience more significant postoperative challenges, such as reperfusion injury or residual PH. Higher RAP values preoperatively can indicate more severe right heart dysfunction, which may complicate the postoperative course. Patients with higher RAP may require more intensive monitoring and supportive care, including longer durations of other interventions, leading to extended hospital stays. Patients with higher preoperative mPAP, RAP, and low TAPSE values may be at a higher risk for reperfusion injury following PTE. Despite successful PTE, some patients may have residual PH or microvasculopathy, which can be more pronounced in those with higher preoperative mPAP and RAP. This residual disease can lead to prolonged recovery times, increased need for postoperative care, and thus longer hospital stays. 

Mortality

Our 30-day, 1-year, and 3-year mortality rates are 0%, 10%, and 7.5%, respectively. This is much lower compared to a recent systematic review that published mortality rates from 55 single centers [11], especially considering our center performs, on average, 4.4 PTEs per year. Lower mortality rates could be attributed to our unique institutional protocols or rigorous patient selection criteria. Investigating these factors further could provide insights into best practices.

Thistlethwaite et al. showed that overall survival was lower in patients with pulmonary artery pressures exceeding 100 mmHg, and failure to lower pulmonary artery pressure during the operation was a strong predictor of in-hospital mortality [25]. In another study, preoperative NT-proBNP levels greater than 1,000 pg/mL were associated with mortality rates nearly four times higher than those with lower NT-proBNP [26]. Our study, however, was unable to find a significant association between these biomarkers, hemodynamic variables, or surgery efficiency on mortality. The lack of significant associations with biomarkers or hemodynamic variables could be due to sample size limitations or variability in patient responses. Future studies should explore these associations with larger cohorts.

Limitations

Despite CTEPH being a rare disease, we were able to include a relatively large cohort in our study. The low representation of certain racial groups, such as American Indian and Alaska Native individuals (2.5%), in our study, is acknowledged as a limitation, which may affect the generalizability of our findings to these populations. With other covariates such as age, preoperative hemodynamics, WHO functional class, and level of disease, we attempted to adjust prolonged circulatory arrest time on oxygen requirements at discharge. The sample size of our study did not support this logistic regression model since each covariate would require at least ten subjects in each outcome group (requiring oxygen at discharge and not requiring oxygen at discharge) to be estimated. Many of our patients were referrals and have incomplete preoperative data. Further, patients who have improved substantially following PTE may not have felt the need for further follow-up.

The limitations of the logistic regression model, such as potential overfitting due to small sample sizes, can impact the study's findings by reducing the generalizability of the results. Future studies could overcome this by increasing the cohort size or employing alternative statistical methods like machine learning approaches. Missing data was addressed analytically through imputation methods. However, patients not returning for follow-up due to improvements might introduce bias, as these cases could skew long-term outcome assessments. Future studies could improve follow-up rates through remote monitoring and structured follow-up protocols. We also did not identify patients who required pulmonary vasodilator therapy prior to surgery nor investigated patients who required prolonged postoperative pulmonary vasodilator therapy or BPA in patients with residual PH, which might be related to prolonged oxygen dependence. The sequential use of PTE followed by BPA may allow for a tailored approach, addressing both proximal and distal obstructions. Inhaled pulmonary vasodilators, such as nitric oxide or prostacyclin analogs, are known to be particularly beneficial immediately post-op when the pulmonary vasculature is adjusting to the removal of obstructions. There is no clear evidence that intravenous pulmonary vasodilator therapy would have a positive impact on postoperative outcomes in our patients with mPAP>46 mmHg, as found by Sakurai et al. in a similar study [14]. The absence of data on preoperative and postoperative pulmonary vasodilator therapy limits understanding of its clinical implications. This would be a useful inquiry as the need for preoperative/postoperative vasodilators or BPA may also reflect pulmonary microvasculopathy. Future studies might investigate the relationship between pulmonary vasodilator therapy, oxygen dependence, and hemodynamic outcomes to better contextualize its role. 

Tools to assess the quality of life for patients with CTEPH are underutilized in clinical practice. Similarly, we were unable to robustly collect subjective data from items such as the St. George's Respiratory Questionnaire. Although we evaluated objective measures of quality of life (i.e., 6MWT), conclusions drawn from this test lack validity due to low completion rates. The underutilization of quality-of-life tools can be addressed by incorporating validated questionnaires such as the 36-item short-form survey instrument (SF-36) or EuroQol-5 Dimension (EQ-5D) in future studies [27]. 

In this context, descriptive analysis involves summarizing and organizing the data to highlight key characteristics and trends. Importantly, the study focused on identifying associations rather than establishing causality. The retrospective nature of the study further limits its ability to infer causality, as it relies on pre-existing data rather than experimental manipulation or prospective observation. As a result, while the findings can generate hypotheses and guide future research, they should not be interpreted as evidence of a cause-and-effect relationship.

## Conclusions

Several hemodynamic parameters were improved by PTE. PTE also reduced oxygen requirements at discharge and one year after surgery. Longer hospital stays were also associated with high-risk hemodynamic profiles (TAPSE, RAP, mPAP), preoperative oxygen requirements, and discharge oxygen requirements were associated with longer intraoperative circulatory arrest times. Clinicians must identify risk factors accurately in order to weigh the benefits and risks of surgery and help intensivists identify high-risk patients. Personalizing management strategies could involve tailoring vasodilator therapy, closely monitoring hemodynamic changes, and utilizing telemedicine for follow-up. Future research should focus on optimizing patient selection criteria through predictive modeling, refining surgical techniques to reduce circulatory arrest times and postoperative management, and exploring the long-term benefits of adjunctive therapies like BPA.
